# Epigenetic Regulation of Sebaceous and Meibomian Glands: From Development to Disease

**DOI:** 10.3390/biomedicines14020468

**Published:** 2026-02-20

**Authors:** Xuming Zhu, Sixia Huang

**Affiliations:** 1 Black Family Stem Cell Institute, Icahn School of Medicine at Mount Sinai, New York, NY 10029, USA; 2 Institute for Regenerative Medicine, Icahn School of Medicine at Mount Sinai, New York, NY 10029, USA; 3 Department of Stem Cell Biology and Regenerative Medicine, Icahn School of Medicine at Mount Sinai, New York, NY 10029, USA; 4 Department of Dermatology, University of Pennsylvania, Philadelphia, PA 19104, USA; sixia@pennmedicine.upenn.edu

**Keywords:** sebaceous gland, Meibomian gland, development, homeostasis, epigenetics, non-coding RNA, stem cells

## Abstract

Sebaceous glands (SGs) and their specialized subtype, Meibomian glands (MGs), play essential roles in skin and ocular surface homeostasis by producing lipids that maintain barrier integrity and stabilize the tear film. Dysregulation of SG and MG biology contributes to a spectrum of disorders, ranging from benign hyperplasia to sebaceous carcinoma and age-related MG dysfunction. Accumulating evidence highlights the importance of epigenetic regulation, including histone modifications, DNA methylation, and non-coding RNAs (ncRNAs), in controlling SG and MG development, homeostasis, and disease susceptibility. Notably, histone modifiers and ncRNAs modulate acinar differentiation, lipid synthesis, and progenitor cell function. Despite these advances, many epigenetic mechanisms, such as histone lactylation, sumoylation, and phosphorylation, remain underexplored, and several common SG/MG disorders, including chalazion and seborrhea, lack mechanistic studies at the epigenetic level. This review synthesizes current knowledge on SG and MG biology, emphasizing epigenetic regulation, and highlights critical gaps to guide future research aimed at improving the understanding and treatment of SG- and MG-related disorders.

## 1. Introduction

Epigenetic regulation represents a critical layer of gene control that integrates multiple factors, such as environmental signals, metabolic state, lifestyle influences, and developmental cues, to shape cellular identity without altering the DNA sequence [1,2,3,4]. In contrast to transcriptional or translational regulation alone, epigenetic mechanisms, including chromatin remodeling, histone modifications, DNA methylation, and non-coding RNA (ncRNA)-mediated processes, provide stable yet reversible control over lineage commitment and cell-state transitions [5,6,7]. This regulatory framework is particularly relevant for tissues and organs characterized by continuous turnover and differentiation, such as the intestine and epidermis [8,9]. Similarly, sebaceous glands (SGs), commonly known as oil-producing glands associated with hair follicles, are holocrine glands with rapid turnover that require tightly coordinated regulation of proliferation, lipid synthesis, and terminal sebocyte maturation to maintain homeostasis [10]. Epigenetic regulation, therefore, may represent a key layer of control in SG biology. While the roles of transcription factors and signaling pathways in SGs have been extensively studied [11], the epigenetic programs that establish, reinforce, or remodel these cellular states in SGs remain underexplored. Importantly, epigenetic pathways operate at the interface of development, regeneration, and disease, and many epigenetic regulators are considered pharmacologically tractable [12]. Elucidating these mechanisms in SG biology not only advances our understanding of tissue-specific gene regulation but also provides a conceptual and therapeutic framework for disorders affecting these glands.

## 2. Overview of the SG and Its Subtypes

SGs, commonly known as oil-producing glands associated with hair follicles, are holocrine glands that secrete sebum to lubricate the skin surface [13]. Mature SGs are maintained by the continuous proliferation of peripheral basal progenitor/stem cells, which undergo stepwise differentiation. During this process, differentiating sebocytes migrate toward the center of the gland and progressively accumulate lipids, including triglycerides, free fatty acids, wax esters, squalene, and cholesterol esters [10]. Once fully differentiated, sebocytes enter the central necrotic zone, rupture, and release their cellular contents. The resulting sebum flows through a short duct connected to the hair follicle canal and eventually reaches the skin surface, where it moisturizes and protects both the skin and hair shaft [14]. The function of SGs is regulated by a complex network of intrinsic factors, such as hormones and aging [15,16], as well as extrinsic influences, including bacterial colonization and ultraviolet (UV) exposure [17,18]. Dysregulated SG activity underlies a spectrum of skin diseases of varying severity, ranging from acne vulgaris and benign SG hyperplasia to aggressive SG carcinoma (SGC) [19,20,21].

**Figure 1:**
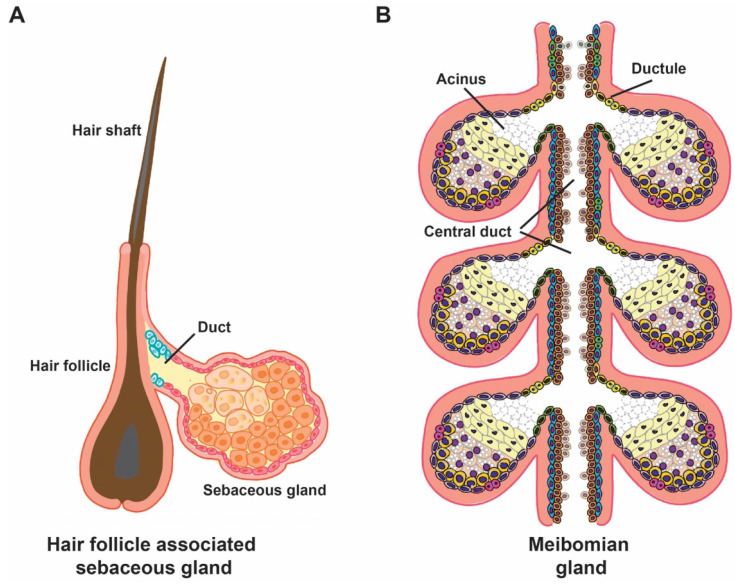
Schematic structure of hair follicle-associated SGs and MGs. The diagrams illustrate the anatomical association of SGs with hair follicles (**A**) and MGs in the tarsal plate (**B**).

Hair follicle-associated SGs are distributed throughout most of the human body, except on the hairless palms, soles, and dorsum of the feet [22]. In addition, several specialized free SGs exist independently of hair follicles. In humans, these include the Meibomian glands (MGs) in the upper and lower eyelids [23], the clitoral glands in females, the preputial glands in males located around the corona and inner foreskin [24], and Montgomery’s glands surrounding the nipples [25]. Among these, MGs are the most extensively studied because their dysfunction leads to evaporative dry eye disease (EDED), a common ocular condition that disproportionately affects older individuals [26,27]. Moreover, MG carcinoma (MGC) represents the majority of all SGC cases [28]. Figure 1 shows schematics of hair follicle-associated SG and MG structures.

Each MG consists of multiple acini that connect to a central duct through short ductules. The central duct is lined by stratified squamous epithelium composed of basal and suprabasal layers [29,30]. Like SGs, MG acini are maintained by peripheral basal cells, which proliferate and give rise to meibocytes that undergo sequential differentiation and lipid accumulation. Upon reaching the acinar center, fully differentiated meibocytes disintegrate and release meibum, which flows through the ductules and central duct to the eyelid margin, ultimately reaching the ocular surface [29,30].

MGs share many similarities with SGs. Both secrete lipids in a holocrine manner and rely on constant replenishment of lipid-releasing cells differentiated from proliferating basal cells. Both also originate from epidermal cells, and their development is governed by overlapping signaling pathways [11,31]. Furthermore, recent evidence indicates that the stem cells maintaining these glands express similar marker genes [32]. However, MGs also exhibit distinct features. For example, meibum differs in lipid composition from sebum [33], and MGs are more densely innervated than SGs [32]. Despite these insights, our understanding of the mechanisms governing the development, homeostasis, and pathological alterations of both hair follicle-associated and specialized SGs, particularly at the epigenetic levels, remains limited. In the following sections, we summarize current knowledge on the epigenetic regulation of the development, adult homeostasis, and disease of hair follicle-associated SGs and MGs.

## 3. Overview of Epigenetic Regulatory Mechanisms

Epigenetic regulation encompasses heritable and reversible changes in gene activity that arise independently of DNA sequence alterations. Such regulation is mediated through diverse molecular processes, including DNA cytosine methylation and hydroxymethylation; covalent modifications of histone proteins; ATP-driven remodeling of chromatin; higher-order chromatin organization; spatial genome interactions in three dimensions; regulatory ncRNA species, such as microRNAs (miRNAs), circular RNAs (circRNAs), and long noncoding RNAs (lncRNAs); and RNA methylation [34,35]. Together, these interconnected layers establish chromatin states that determine transcriptional permissiveness and gene regulatory potential. In this section, we provide an overview of the principal epigenetic pathways known or likely to regulate the function, development, and homeostasis of SGs and their subtypes.

### 3.1. DNA Methylation

DNA methylation represents the most extensively studied epigenetic modification and involves the covalent addition of a methyl group (CH_3_) to cytosine residues, primarily within gene regulatory regions such as promoters. This modification occurs predominantly at cytosine–phosphate–guanine (CpG) dinucleotides, which are often clustered into CpG islands spanning approximately 0.5–2 kb and are present in the promoter region in about 70% of human genes [36,37]. Methylation at the 5-carbon position of cytosine generates 5-methylcytosine (5mC), a mark generally associated with transcriptional repression and stable gene silencing [38].

DNA methylation patterns are established and maintained by DNA methyltransferases (DNMTs), among which DNMT1, DNMT3A, and DNMT3B have been extensively characterized. DNMT1 is primarily responsible for maintaining preexisting methylation marks during DNA replication by recognizing hemi-methylated DNA, whereas DNMT3A and DNMT3B function as de novo methyltransferases that introduce new methylation patterns during development and cellular differentiation [39,40].

### 3.2. Covalent Modifications of Histone Proteins

#### 3.2.1. Methylation

Histone methylation is a reversible epigenetic modification in which one, two, or three methyl groups are added to specific lysine or arginine residues on histone proteins, especially within the N-terminal tails of histones H3 and H4 [41]. By altering chromatin architecture, this modification regulates DNA accessibility and thereby modulates transcriptional activity [42]. The functional outcome of histone methylation is highly context dependent, as it can either promote gene activation or repression depending on the modified residue and the extent of methylation [43,44].

Histone methylation is catalyzed by histone methyltransferases (HMTs), while removal of methyl groups is mediated by histone demethylases (HDMs), allowing dynamic regulation of chromatin states [45,46]. This modification plays essential roles in diverse biological processes, including transcriptional control, DNA damage repair, cell cycle progression, development, and disease pathogenesis [47,48,49].

The biological consequences of histone methylation depend on the specific amino acid residues involved. Methylation at histone H3 lysine 4, 36, and 79 (H3K4, H3K36, and H3K79), as well as histone H4 lysine 20 (H4K20), is generally associated with transcriptional activation. In contrast, trimethylation of histone H3 lysine 9 or lysine 27 (H3K9me3 or H3K27me3) is strongly linked to transcriptional repression and long-term gene silencing [46,50,51,52,53,54].

#### 3.2.2. Acetylation

Histone acetylation is a core epigenetic modification that regulates chromatin structure and gene transcription through the reversible addition of acetyl groups to lysine residues within histone tails [55]. This process is catalyzed by histone acetyltransferases (HATs), which neutralize the positive charge of lysine residues, weaken histone–DNA interactions, and promote an open euchromatic state. Consequently, regulatory DNA regions become more accessible to transcription factors and RNA polymerase, facilitating transcriptional activation [56,57].

HATs are broadly divided into two functional categories. Type A HATs are nuclear enzymes that acetylate nucleosomal histones and other chromatin-associated proteins, thereby directly influencing transcription. In contrast, type B HATs are cytoplasmic enzymes that acetylate newly synthesized histones before chromatin assembly and do not directly regulate transcription [58,59]. Type A HATs are further classified into five families based on homology and acetylation mechanisms: the GNAT family (GCN5, PCAF, ELP3); the p300/CBP family (p300, CBP); the MYST family (MOZ, MORF, TIP60, HBO1, SAS2); nuclear receptor coactivators (e.g., SRC-1); and general transcription factors (e.g., TAF1, TFIIC90) [60].

Conversely, histone deacetylases (HDACs) remove acetyl groups from histone tails, restoring positive charge and strengthening histone–DNA interactions, thereby promoting chromatin compaction and transcriptional repression [61]. HDACs are classified into four groups (Classes I–IV) based on homology to yeast enzymes. Classes I, II, and IV are zinc-dependent, whereas Class III HDACs (sirtuins) require NAD^+^ for catalytic activity [62,63]. Among these, Class I HDACs are the most extensively studied. HDAC1 and HDAC2 are closely related and frequently function redundantly, associating with repressive complexes such as NuRD, Sin3A, CoREST, and MiDAC [64]. In contrast, HDAC3 primarily interacts with the SMRT/N-CoR corepressor complex, suggesting regulation of a distinct set of target genes [65,66].

Working together, HATs and HDACs maintain a dynamic and balanced acetylation landscape, which is essential for the precise regulation of gene expression across diverse cellular processes. Dysregulation of histone acetylation has been implicated in numerous diseases [62,67]. Pharmacological agents targeting this process, such as HDAC inhibitors, have already been approved for the treatment of several cancers [68,69], and additional compounds are currently undergoing clinical evaluation, highlighting the therapeutic potential of modulating histone acetylation [63,70].

### 3.3. NcRNAs

NcRNAs are RNA transcripts that do not encode proteins but play essential roles in epigenetic regulation and genome organization. By interacting with DNA, RNA, and chromatin-associated proteins, ncRNAs modulate gene expression, chromatin accessibility, and transcriptional programs. Major ncRNA classes include ribosomal RNAs (rRNAs), transfer RNAs (tRNAs), miRNAs, circRNAs, and lncRNAs, each contributing to distinct regulatory layers [71,72].

#### 3.3.1. MiRNAs

MiRNAs are small ncRNAs of approximately 21–23 nucleotides that regulate gene expression post-transcriptionally by promoting translational repression or mRNA degradation. Since their discovery nearly three decades ago in Caenorhabditis elegans and their recognition in 2001 as a conserved RNA class across higher eukaryotes, miRNAs have been established as fundamental regulators controlling diverse biological processes [73].

Most miRNAs are transcribed as long primary transcripts (pri-miRNAs) containing stem–loop structures, typically originating from non-coding regions or introns. Canonical miRNA biogenesis involves sequential processing by two RNase III enzymes. In the nucleus, the Drosha–DGCR8 Microprocessor complex cleaves pri-miRNAs to produce precursor miRNAs (pre-miRNAs), which are subsequently exported to the cytoplasm and processed by Dicer into a miRNA duplex. One strand is selectively loaded into an Argonaute protein to form the RNA-induced silencing complex (RISC), while the passenger strand is discarded. The mature miRNA then guides RISC to complementary RNA targets to mediate gene silencing. In addition, non-canonical Drosha- or Dicer-independent pathways further expand miRNA regulatory diversity [73,74,75].

Through fine-tuning gene expression networks, miRNAs play critical roles in development, homeostasis, and pathogenesis [76,77,78]. Their stability, specificity, and broad regulatory capacity also make them valuable biomarkers and promising therapeutic targets within epigenetic and post-transcriptional regulatory frameworks [79,80,81].

#### 3.3.2. LncRNAs

LncRNAs are a heterogeneous class of RNA transcripts longer than 200 nucleotides that lack protein-coding capacity but play critical roles in gene regulation and cellular homeostasis. Transcribed by RNA polymerases I, II, or III, or derived from processed introns, lncRNAs regulate diverse biological processes, including development, differentiation, metabolism, and disease pathogenesis, particularly cancer [82,83]. Despite their functional importance, lncRNA annotation remains challenging due to their numerous isoforms, overlapping genomic organization, and rapid evolutionary divergence compared with protein-coding genes [84,85].

Many lncRNAs are expressed in a cell type-specific manner and exert epigenetic functions by interacting with chromatin-modifying complexes, regulating enhancer activity, and shaping higher-order chromatin architecture [86]. Some lncRNAs participate in the formation of nuclear condensates through phase separation, linking their expression to spatial and temporal control of gene transcription during development [87,88]. Beyond the nucleus, lncRNAs also operate in the cytoplasm, where they influence mRNA translation, stability, metabolism, and intracellular signaling pathways [89,90].

Functionally, lncRNAs often exhibit modular structures and are enriched in repetitive elements that facilitate interactions with DNA, RNA, and proteins [82,91]. Through these interactions, lncRNAs act as molecular scaffolds, guides, decoys, or sponges, regulating gene expression at transcriptional and post-transcriptional levels [92]. Notably, lncRNAs engage in extensive crosstalk with other ncRNAs, such as miRNAs, and with mRNAs, forming competing endogenous RNA (ceRNA) networks that fine-tune RNA processing, stability, and translation [93,94]. Collectively, these properties position lncRNAs as key regulators within complex epigenetic and gene regulatory networks.

## 4. Overview of SG/MG Development

### 4.1. SG Development

The embryonic development of the SG is closely linked to the development and differentiation of the hair follicle and the epidermis. In humans, SG development begins between weeks 13 and 16 of fetal life. New SGs do not typically form postnatally; instead, existing glands increase in size with age [95]. In mice, the SG is the last epithelial lineage to emerge during hair follicle morphogenesis, appearing at the bulbous peg stage and remaining permanently associated with the upper region of the hair follicle. The first sebocytes become detectable shortly after birth, as indicated by the expression of specific marker molecules. In certain hair follicle types, two prominent SGs originate from a single small cluster of sebocytes. Once this population reaches a critical size, it divides into two clusters that subsequently mature into fully differentiated glands [11].

### 4.2. MG Development

During early development, the MG anlage closely resembles that of the hair follicle and is therefore often described as a “hair follicle without a hair shaft.” [29] In humans, MG development occurs between the third and seventh months of gestation during the sealed-lid phase of eyelid development. Histological analyses of human fetuses have shown that MG anlages first appear around the 80 mm crown–rump length stage as localized epithelial downgrowths from the junctional epithelium, with upper eyelid development preceding the lower. These downgrowths extend slowly into the mesenchyme and are initially associated with a mesodermal cap characterized by enzymatic activity indicative of active epithelial–mesenchymal interactions [31,96].

By the 142 mm stage, secondary epithelial outgrowths emerge, marking the onset of lipid production. Lipid droplets first appear in central cells and progressively enlarge and spread toward the eyelid margin, accompanied by metabolic and enzymatic changes consistent with meibocyte differentiation. As development proceeds, multiple outgrowths form along the epithelial cord, which ultimately give rise to lipid-producing alveoli. Differentiation of the MG duct follows, with the appearance of keratohyalin granules and early keratinization [31,96].

In mice, MG development begins at embryonic (E) day 18.5 with the formation of epithelial placodes in the fused eyelids, accompanied by mesenchymal condensation. Most subsequent development occurs postnatally. Epithelial invagination and elongation take place between birth and postnatal (P) day 3, followed by ductal lumen formation. Acinar differentiation and ductal branching are evident by P5, and distinct ductal and acinar structures are established by P8. Morphological development is largely complete by P15, shortly after eyelid opening. However, lipidomic analyses indicate that full functional maturation of the MG, as reflected by adult-like meibum composition, is not achieved until approximately P21 [97,98].

## 5. Adult SG/MG Homeostasis

### 5.1. SG Homeostasis

SG homeostasis is maintained through continuous cellular renewal that is tightly integrated with hair follicle biology. Accumulating experimental evidence indicates that SG maintenance depends on coordinated interactions among multiple stem and progenitor cell populations within the pilosebaceous unit. Disruption of one compartment frequently compromises the other, underscoring their functional interdependence during both homeostasis and regeneration [11,99].

Two non-mutually exclusive models have been proposed to explain SG renewal. One model posits that unipotent, lineage-committed progenitor cells located at the periphery of the SG sustain gland turnover under homeostatic conditions. The alternative model suggests that multipotent hair follicle stem cells are mobilized to regenerate the SG, particularly in response to stress or injury [11,99].

Strong support for the local progenitor model comes from a lineage-tracing study showing that a subset of Slc1a3^+^ basal cells residing at the SG periphery can sustain long-term renewal of the entire gland. This finding reinforces the concept that the SG functions as a self-maintained stem cell niche [100]. In parallel, additional lineage-tracing studies demonstrate that several hair follicle stem cell populations possess sebaceous differentiation potential. Keratin 15^+^ bulge stem cells and Lgr6^+^ isthmus stem cells can generate sebocytes, and bulge-derived progeny can replenish SG stem and progenitor pools [101,102].

The junctional zone (JZ) of the hair follicle represents another key fate-determining niche for SG maintenance. Lrig1^+^ keratinocytes located in this region are among the best-characterized SG-associated stem cells and are sufficient for long-term gland renewal during homeostasis. Clonal analyses reveal neutral competition among Lrig1^+^ progenitors, resulting in progressive monoclonality within the SG. These cells exhibit dual fate potential, contributing either to sebocytes or to the sebaceous duct and infundibulum [103,104].

Collectively, these observations suggest that SG homeostasis is maintained by multiple stem cell reservoirs, with local SG progenitors supporting steady-state renewal and hair follicle-derived stem cells providing regenerative flexibility. Crosstalk among these compartments ensures robust maintenance and adaptive regeneration of the pilosebaceous unit.

### 5.2. MG Homeostasis

Current evidence indicates that MG homeostasis is maintained by rare, spatially restricted stem and progenitor cell populations organized within distinct glandular compartments. Early label-retaining cell studies suggested the presence of slow-cycling stem cells in both acinar and ductal regions [105,106]; however, genetic lineage-tracing approaches have since provided a clearer and more refined framework.

Lineage-tracing studies using Keratin 14-Cre-driven reporters demonstrate that MG acini and ducts are largely sustained by distinct stem cell pools. Individual acini are clonally derived, indicating long-term self-renewal from local acinar stem cells, whereas the ductal epithelium is maintained independently [106]. Although definitive MG stem cell markers remain elusive, several candidate populations have been implicated. Slc1a3^+^ basal cells selectively replenish acini, supporting the concept of a locally self-maintained stem cell niche. In contrast, Lrig1^+^, Lgr6^+^, Axin2^+^, Gli2^+^, Krt17^+^, and Krox20^+^ cells contribute to both acinar and ductal compartments [32,107,108], highlighting functional heterogeneity among MG progenitors.

Recent transcriptomic and lineage-tracing analyses further identify the ductules as an important integrative niche, with cells exhibiting mixed ductal and acinar gene signatures and contributing to both lineages [32]. Live-imaging-based lineage tracing in MG explants provides additional evidence that ductular stem cells can migrate toward the acinar periphery, offering a mechanistic explanation for how ductular progenitors contribute to acinar homeostasis [32]. While stem cells appear to behave predominantly as unipotent populations under homeostatic conditions, these findings suggest potential plasticity that may enable restoration of gland structure following injury or stress. Whether such plasticity operates under physiological conditions remains to be determined by future in vivo lineage-tracing studies, potentially using two-photon imaging approaches.

Together, these data support a model in which MG homeostasis is sustained by multiple, locally acting stem and progenitor populations with compartment-biased functions, coordinated through intercompartmental crosstalk and context-dependent plasticity, closely paralleling principles established for hair follicle-associated SG maintenance.

## 6. Common SG and MG Diseases

### 6.1. Acne Vulgaris

Acne vulgaris is a common, chronic inflammatory disorder of the pilosebaceous unit that mainly affects adolescents but may persist into or arise during adulthood [109]. Although not life-threatening, acne can cause permanent scarring, post-inflammatory hyperpigmentation, and significant psychosocial distress [110]. Disease prevalence and severity vary with age, sex, and ethnicity, with adolescent acne more common in males and adult acne predominating in females [111].

The pathogenesis of acne is multifactorial and classically involves four interrelated processes: androgen-driven SG hyperactivity and excess sebum production, follicular hyperkeratinization leading to micro-comedo formation, dysbiosis and proliferation of *Cutibacterium acnes*, and activation of inflammatory immune responses [110,111]. Central to acne pathogenesis is SG dysfunction, in which androgen-driven sebocyte hyperactivity and excessive sebum production create a permissive microenvironment for subsequent pathological events. Thus, acne preferentially develops in sebaceous-rich, hormonally responsive regions such as the face, chest, and upper back [112]. Disease expression and severity are modulated by genetic predisposition, hormonal fluctuations, insulin resistance, and environmental and lifestyle factors, including occlusion, cosmetic use, mechanical irritation, psychological stress, medications, and diet [111].

Management of acne requires long-term, individualized therapy aimed at suppressing lesion formation and preventing scarring rather than achieving immediate cure. Patient education, gentle skincare practices, and maintenance treatment, most commonly with topical retinoids and antibiotics, are essential [113,114]. With appropriate intervention, prognosis is generally favorable, although the lasting psychosocial and cosmetic impact underscores the importance of early and effective disease control [115].

### 6.2. SG Neoplasms

SG neoplasms reflect dysregulated sebocyte proliferation and differentiation along a biological continuum that spans non-neoplastic enlargement to overt malignancy. This spectrum includes sebaceous hyperplasia, sebaceous adenoma, sebaceoma, and SGC. Sebaceous hyperplasia represents the most common manifestation and is characterized by expansion of otherwise structurally and functionally normal SGs. In contrast, true sebaceous neoplasms are rare and occur predominantly in older individuals [116]. While multiple sebaceous neoplasms are strongly associated with Muir–Torre syndrome [117], sebaceous hyperplasia is more closely linked to chronic UV exposure and age-related alterations in SG homeostasis [118,119].

Sebaceous hyperplasia is defined by increased lobule number and gland size with preservation of normal architecture, polarity, and sebocyte maturation. These features distinguish it from neoplastic lesions and underscore its classification as a disorder of SG growth rather than transformation. Although biologically benign and unrelated to mismatch repair deficiency, its predilection for facial skin and tendency toward multiplicity confer a significant cosmetic burden, and recurrence is common following destructive therapies [20,120].

Sebaceous adenoma and sebaceoma represent benign neoplastic counterparts marked by altered sebocyte differentiation and expansion of basaloid progenitor populations within the SG. Despite their indolent clinical behavior, histopathologic evaluation is essential to delineate their position along the SG differentiation hierarchy and to exclude well-differentiated SGC [121]. Importantly, these lesions may serve as cutaneous indicators of systemic defects in DNA mismatch repair, necessitating consideration of Muir–Torre syndrome and appropriate oncologic surveillance [122].

SGC represents the malignant extreme of SG dysregulation, arising from aberrant sebocytic differentiation coupled with invasive growth. The majority of SGCs occur in the periocular region, particularly the eyelids, which harbor a high density and diversity of SGs, including MGs, glands of Zeis (associated with eyelash hair follicles), caruncular glands, SGs of the eyelid hair follicles, and glands of the eyebrows [123]. MGs give rise to most periocular SGCs, leading to the designation MGC, and likely accounting for the increased incidence of SGC at this site due to their high abundance [123]. Approximately one quarter of cases arise at extraocular locations, most commonly in the head and neck, with a smaller proportion occurring in other hair-bearing regions, including the genitalia [124]. Although clinical presentation, management, and prognosis are broadly similar across anatomic sites [124], periocular SGCs exhibit greater local aggressiveness, higher recurrence rates, and an increased risk of metastasis compared with extraocular tumors [125]. Risk factors such as advanced age, immunosuppression, prior irradiation, and mismatch repair deficiency are linked to SGC pathogenesis [21]. Complete surgical excision with histologic margin control remains essential, and increased recognition of SG-derived malignancies, particularly in periocular locations, has improved outcomes despite ongoing morbidity [126].

### 6.3. MG Dysfunction (MGD) and EDED

EDED is the predominant form of dry eye disease (DED), primarily caused by MGD. In EDED, insufficient or altered meibum leads to tear film instability, accelerated evaporation, ocular surface inflammation, and irritation [127]. MGD is the most frequent underlying cause of EDED and may coexist with or mimic aqueous-deficient DED, making accurate diagnosis critical for targeted management [128].

MGD is a heterogeneous condition, classified as low-delivery (obstructive or hyposecretory) or high-delivery (hypersecretory) based on meibum output [129]. Obstructive MGD, the most common form, results from hyper-keratinization of the gland ducts, gland dropout, or age- and hormone-related changes, whereas hypersecretory MGD is associated with rosacea or seborrheic dermatitis [130]. Both forms alter meibum composition, reducing lipid quality and destabilizing the tear film. Stasis of meibum promotes bacterial growth and lipase activity, generating free fatty acids that exacerbate inflammation and gland obstruction, creating a self-perpetuating “vicious circle” linking MGD to EDED [131].

The global prevalence of MGD is approximately 36%, which varies geographically, from 60.8–71% in Asian populations to 16.4–42.7% in Caucasians, and is most common in older adults [132]. Diagnosis relies on clinical assessment and specialized tests, while management is individualized. Conventional therapy focuses on relieving obstruction with warm compresses, lid massage, and lid hygiene, whereas newer interventions, such as vectored thermal pulsation and intense pulsed light therapy, may improve outcomes [133].

## 7. Epigenetic Regulation of SG and MG Development, Homeostasis, and Disease

Accumulating evidence highlights epigenetic regulation as a central mechanism governing SG and MG development, maintenance, and pathological remodeling. Multiple histone-modifying enzymes have been shown to fine-tune sebocyte differentiation, lipid synthesis, and glandular architecture. Disruption of histone H3K36 methylation results in hypertrophic SGs and MGs, with increases in both gland size and number, underscoring its role in restraining gland expansion [134]. In line with this, epithelial deletion of the histone methyltransferase SETD8, acting downstream of c-MYC, leads to the progressive loss of long-lived epidermal progenitors and irreversible SG depletion, indicating a requirement for precise epigenetic control in gland maintenance [135]. Likewise, hypomorphic mutation of *Ash1l*, another histone methyltransferase essential for epidermal homeostasis, causes SG enlargement in mice [136], further linking dysregulated histone methylation to gland hyperplasia.

Histone acetylation also plays a key role in sebocyte metabolism and gland homeostasis. In human sebocytes, HDAC1 suppresses lipogenesis by inhibiting SREBP1 transcription, thereby limiting lipid accumulation [137]. Pharmacological studies further support this axis, showing that minocycline, widely used in acne treatment, inhibits the histone acetyltransferase p300 in a dose-dependent manner without affecting global HDAC activity, resulting in reduced histone acetylation, suppressed SREBP1 expression, and decreased lipid production. Conversely, p300 overexpression enhances histone acetylation and sebocyte lipogenesis, positioning p300 as a critical epigenetic regulator of SG function [138]. In vivo, loss of the X-linked histone demethylase UTX (KDM6A) in epithelial cells leads to genome-wide reductions in H3K27 acetylation with minimal effects on H3K27me3, driving enlargement of both SG and MG in female mice and highlighting the importance of acetylation balance in gland size control [139].

DNA methylation contributes more subtly to SG regulation. Conditional deletion of *Dnmt3a* does not disrupt keratinocyte lineage specification or adult skin homeostasis, including in SGs, although it increases susceptibility to sebaceous adenoma formation [140]. In contrast, Keratin 14-Cre-driven deletion of the maintenance DNA methyltransferase DNMT1 in epidermal tissue of mice results in SG hyperplasia, suggesting that DNA methylation is dispensable for lineage commitment but important for long-term glandular stability [141].

**Figure 2:**
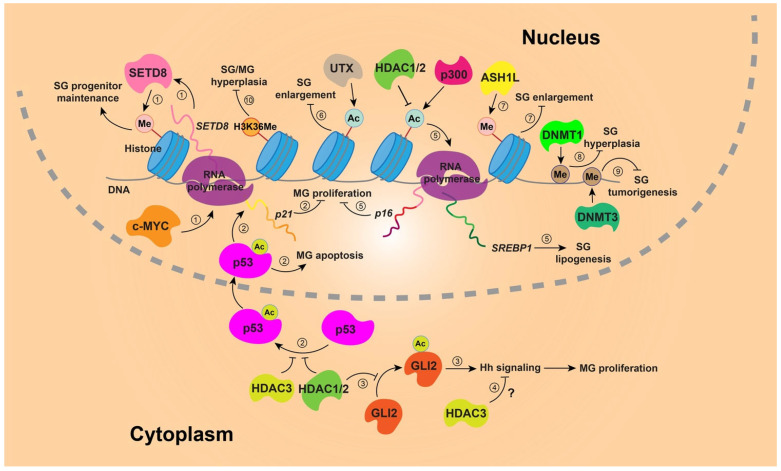
Diagram showing histone-modifying enzymes and DNA modifications in SG and MG. ① c-MYC promotes the transcription of *SETD8* mRNA, and SETD8 protein catalyzes histone methylation, which is required for SG progenitor maintenance. ② Acetylation of p53 enhances its stability and function, allowing it to enter the nucleus and induce *p21* expression, thereby inhibiting MG cell proliferation. p53 also promotes apoptosis in MG cells. HDAC1/2 and HDAC3 remove p53 acetylation to suppress the p53 pathway. ③ HDAC1/2 deacetylate GLI2, a mediator of Hh signaling that maintains MG cell proliferation. ④ HDAC3 also suppresses the Hh pathway through an unknown mechanism in MG cells. ⑤ HDAC1/2 remove histone acetylation to induce *p16* expression, which may inhibit MG proliferation. Meanwhile, HDAC1 and p300 inhibit or promote histone acetylation, respectively, thereby regulating *SREBP1* expression to control lipid synthesis in sebocytes. ⑥ UTX promotes histone acetylation to inhibit SG enlargement. ⑦ ASH1L facilitates DNA methylation to suppress SG enlargement. ⑧ DNMT1 suppresses SG overgrowth by catalyzing DNA methylation. ⑨ DNMT3 reduces SG tumor susceptibility by mediating DNA methylation. ⑩ H3K36 methylation suppresses SG/MG hyperplasia.

Similarly, genetic perturbation of HDACs reveals their vital roles: homozygous loss of HDAC1 and heterozygous deletion of HDAC2 in the epithelium from embryonic stages leads to SG and MG hyperplasia [142,143], whereas combined deletion of HDAC1/2 or loss of HDAC3 in adult MG epithelium causes ductal dilation, acinar atrophy, aberrant proliferation, and increased apoptosis, features consistent with MGD, indicating distinct and non-redundant HDAC functions in gland maintenance [144]. Mechanistically, HDAC1/2 and HDAC3 negatively regulate p53 acetylation; HDAC1/2 also suppress suppresses *p16* expression, whereas HDAC3 exerts its effects in part through repression of Hedgehog (Hh) signaling [144]. Additionally, altered HDAC1/2 activity may account for increased association of GLI2 with acetylated lysine in aged MGs, a key transcription factor for MG homeostasis [32]. Figure 2 outlines histone-modifying enzymes and DNA modifications known to regulate SG and MG biology.

NcRNAs further expand the epigenetic landscape of SG/MG disease. In SGC, multiple miRNAs have been implicated in tumor progression and metastasis. miR-3907 is overexpressed and promotes the proliferation and migration of eyelid SGC cells by targeting Thrombospondin 1 [145]. In contrast, miR-200c and miR-141 are downregulated in eyelid SGC, with reduced expression correlating with larger tumor size, poor differentiation, lymph node metastasis, and shorter disease-free survival, likely through modulation of epithelial–mesenchymal transition via E-cadherin and ZEB2 [146]. Similarly, miR-651-5p is reduced in SGC, and its restoration suppresses UV radiation-induced malignant behaviors by targeting ZEB2 [147].

Beyond malignancy, miRNAs also contribute to inflammatory sebaceous disorders. miR-146a has been implicated in acne pathogenesis by modulating inflammatory signaling, promoting sebocyte proliferation, and indirectly enhancing lipid production through downregulation of GNG7 [148].

**Figure 3:**
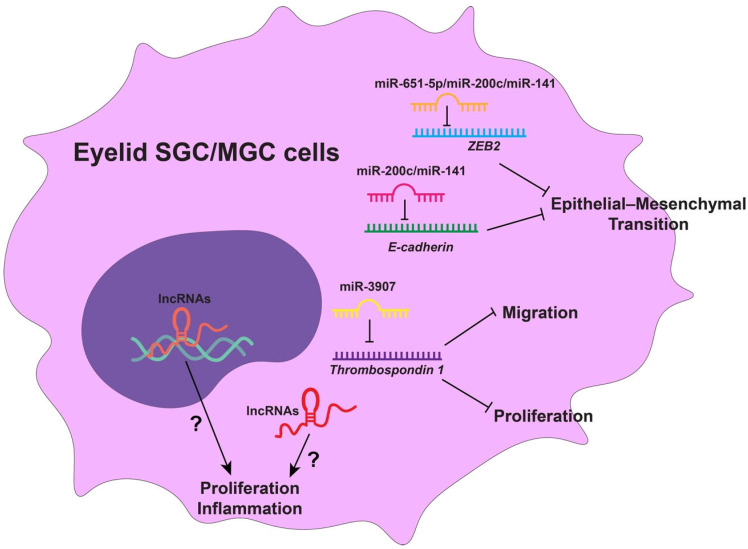
NcRNAs in eyelid SG and MG tumorigenesis. miR-3907 is overexpressed in eyelid SGC, promoting cell proliferation and migration by targeting *Thrombospondin 1*. miR-200c and miR-141 function as tumor suppressors by regulating epithelial–mesenchymal transition via *E-cadherin* and *ZEB2*. miR-651-5p inhibits UV radiation-induced malignant behaviors in eyelid SGC through *ZEB2*. Additionally, multiple lncRNAs are differentially expressed in MGC, potentially contributing to inflammatory signaling and tumor cell proliferation.

At a broader level, transcriptomic analyses have identified hundreds of differentially expressed lncRNAs in MGC, many of which are linked to inflammatory pathways and tumor cell proliferation [149], suggesting additional layers of epigenetic control in SG/MG pathology. Figure 3 summarizes ncRNAs known or potentially involved in eyelid SG and MG tumorigenesis.

## 8. Epigenetic Regulation of PPARγ

Direct evidence linking epigenetic mechanisms to SG biology remains emerging. Given the central role of PPARγ in SG/MG development, metabolism, homeostasis, and diseases [11,16,150,151,152,153,154], insights from adipose and mesenchymal systems provide a valuable framework for understanding how epigenetic regulation of PPARγ may shape SG and MG biology.

Multiple layers of chromatin-based regulation converge on the *PPARG* locus to control its transcriptional competence. PTIP, a cofactor associated with histone H3K4 methyltransferase complexes, is required for PPARγ induction during preadipocyte differentiation, where its loss leads to diminished H3K4me3 and reduced RNA polymerase II occupancy at the *PPARG* promoter [155]. DNA methylation also contributes to PPARγ repression, as hypermethylation of *PPARG* has been observed in insulin-resistant adipocytes, with DNMT1 implicated in maintaining these early epigenetic alterations [156]. In contrast, active demethylation mechanisms promote PPARγ activation: the H3K9 demethylase JMJD2B facilitates adipogenesis by relieving repressive chromatin marks at *PPARG* and *C/EBPα* loci [157], while TET-mediated hydroxymethylation at the *Pparg* locus is essential for initiating adipogenic differentiation [158]. More recently, histone lysine lactylation (Kla), a metabolic-driven post-translational modification in which lactate-derived lactyl groups are added to lysine residues on histones [159,160], has emerged as an additional regulatory layer, with lactate-induced H3K18la at the *PPARG* promoter enhancing transcription in bone marrow-derived mesenchymal stem cells [161].

Beyond chromatin modifiers, ncRNAs constitute a major regulatory axis controlling PPARγ expression and function. Multiple miRNAs, including miR-27a/b, miR-130, miR-155, miR-221, miR-222, miR-301a, miR-540, and miR-651, suppress adipogenesis by directly targeting *PPARG* transcripts and associated regulatory pathways [162,163,164,165,166,167,168,169]. In parallel, lncRNAs modulate PPARγ at transcriptional, post-transcriptional, and post-translational levels. LncRNAs such as *H19*, *SNHG3*, *SNHG1*, *U90926*, *NEAT1*, *HOTAIR*, and *TUG1* influence PPARγ expression by remodeling chromatin accessibility, regulating miRNA availability, modulating alternative splicing, or controlling ubiquitination-dependent protein stability [170,171,172,173,174,175,176].

Collectively, these studies establish PPARγ as a convergence point for diverse epigenetic mechanisms, including DNA methylation, histone modification, chromatin remodeling, and ncRNA regulation. Although much of this knowledge derives from adipogenic systems, the shared reliance of sebocytes on PPARγ-driven lipid metabolism strongly suggests that similar epigenetic programs may operate in SG and MG biology. Defining how these regulatory layers are integrated in gland-specific contexts remains an important direction for future investigation.

## 9. Conclusions and Perspectives

SGs and MGs are dynamic epithelial appendages essential for cutaneous and ocular surface homeostasis. Beyond lipid secretion, these glands are metabolically active, stem cell-supported organs whose maintenance depends on coordinated programs of sebaceous differentiation, lipid metabolism, and epithelial renewal. Recent advances in lineage tracing and single-cell profiling have revealed unexpected cellular heterogeneity and regulatory complexity within SGs and MGs, positioning them as instructive models of epithelial plasticity.

Epigenetic regulation is emerging as a central mechanism governing SG and MG development, homeostasis, and disease. Histone modifiers, chromatin regulators, DNA methylation machinery, and ncRNAs collectively shape sebocyte/meibocyte fate decisions and lipid output. Disruption of these pathways contributes to hyperplasia, atrophy, inflammation, and malignant transformation. PPARγ functions as a key transcriptional and metabolic hub integrating epigenetic, hormonal, and environmental cues. While much of our understanding of PPARγ regulation derives from adipogenic systems, growing evidence suggests that similar epigenetic principles operate in SG and MG biology and merit direct investigation in gland-specific contexts.

An emerging and largely unexplored aspect of SG and MG regulation is histone Kla, which directly links metabolism to chromatin state. Human SGs are highly glycolytic and glutaminolytic tissues, supporting sustained lipid synthesis [13,177]. Notably, diabetic patients exhibit elevated serum lactate levels and an increased prevalence and severity of MGD [178,179], suggesting that metabolic alterations may reshape the epigenetic landscape of sebaceous tissues. Given recent evidence that lactate-driven histone Kla modulates differentiation and inflammation in other systems [160,180], defining its role in sebaceous differentiation gene regulation and MG pathology represents a promising direction for future research.

Despite increasing interest, epigenetic mechanisms regulating SG and MG biology remain underexplored, particularly those involving histone modifiers, lncRNAs, and circRNAs. Moreover, additional epigenetic processes, such as SUMOylation, histone and DNA phosphorylation, and other noncanonical chromatin modifications, are not discussed here due to the paucity of available studies, underscoring major knowledge gaps in the field. Similarly, several clinically relevant SG/MG disorders, like chalazion and seborrhea, remain poorly characterized at the epigenetic level and were not addressed owing to limited mechanistic data.

Future integration of single-cell and spatial multi-omics with metabolic profiling and functional models will be essential to unravel how epigenetic programs interface with local and systemic cues in SG and MG biology. Advancing this understanding may enable mechanism-based therapeutic strategies for common disorders such as acne and MGD, as well as for aggressive malignancies, including sebaceous carcinoma.

## Data Availability

No new data were created or analyzed in this study. Data sharing is not applicable to this article.

## Abbreviations

Abbreviation	Explanation
5mC	5-methylcytosine
Ash1l	ASH1-like histone lysine methyltransferase
Axin2	Axis inhibition protein 2
CBP	CREB-binding protein
C/EBPα	CCAAT/enhancer-binding protein alpha
ceRNA	Competing endogenous RNA
circRNA	Circular RNA
c-MYC	MYC proto-oncogene
CoREST	Corepressor of REST
CpG	Cytosine–phosphate–guanine
DED	Dry eye disease
DGCR8	DiGeorge syndrome critical region gene 8
DNMT	DNA methyltransferase
Drosha	Ribonuclease III enzyme Drosha
EDED	Evaporative dry eye disease
ELP3	Elongator acetyltransferase complex subunit 3
GCN5	General control of amino acid synthesis 5
GNG7	G protein subunit gamma 7
Gli2	GLI family zinc finger 2
HDAC	Histone deacetylase
HDAC1	Histone deacetylase 1
HDAC2	Histone deacetylase 2
HDAC3	Histone deacetylase 3
HAT	Histone acetyltransferase
HBO1	Histone acetyltransferase binding to ORC1
H3K18la	Lactylation at lysine 18 (K18) on histone H3
H3K4	Histone H3 lysine 4
H3K36	Histone H3 lysine 36
H3K79	Histone H3 lysine 79
H3K9me3	Trimethylation of histone H3 lysine 9
H3K27me3	Trimethylation of histone H3 lysine 27
H4K20	Histone H4 lysine 20
H19	Long noncoding RNA H19
HBEGF	Heparin-binding EGF-like growth factor
Hh	Hedgehog
HOTAIR	HOX antisense intergenic RNA
JZ	Junctional zone
JMJD2B	Jumonji domain-containing 2B histone demethylase
Kla	Lysine lactylation
KDM6A	Lysine demethylase 6A
Krt17	Keratin 17
Krox20	EGR2, Early Growth Response 2
Lgr6	Leucine-rich repeat-containing G protein-coupled receptor 6
Lrig1	Leucine-rich repeats and immunoglobulin-like domains 1
MG	Meibomian gland
MGC	Meibomian gland carcinoma
MGD	Meibomian gland dysfunction
MiDAC	Mitotic deacetylase complex
miRNA	MicroRNA
MOZ	Monocytic leukemia zinc finger protein
MORF	MOZ-related factor
ncRNA	Non-coding RNA
NEAT1	Nuclear enriched abundant transcript 1
N-CoR	Nuclear receptor corepressor
NuRD	Nucleosome remodeling and deacetylase complex
PCAF	p300/CBP-associated factor
PPARγ	Peroxisome proliferator-activated receptor gamma
PTIP	PAX transactivation-domain interacting protein
pre-miRNA	Precursor miRNA
pri-miRNA	Long primary transcript of microRNA
RISC	RNA-induced silencing complex
SETD8	SET domain-containing protein 8
Sin3A	SIN3 transcription regulator family member A
Slc1a3	Solute carrier family 1 member 3
SNHG1	Small nucleolar RNA host gene 1
SNHG3	Small nucleolar RNA host gene 3
SRC-1	Steroid receptor coactivator-1
SREBP1	Sterol regulatory element-binding protein 1
TAF1	TATA-box binding protein-associated factor 1
TET	Ten-eleven translocation
TIP60	Tat-interactive protein 60 kDa
tRNAs	Transfer RNAs
TUG1	Taurine upregulated gene 1
U90926	Long noncoding RNA U90926
UV	Ultraviolet
ZEB2	Zinc finger E-box binding homeobox 2
SG	Sebaceous gland
SGC	Sebaceous gland carcinoma

## References

[1] Alegría-Torres J.A., Baccarelli A., Bollati V. (2011). Epigenetics and lifestyle. Epigenomics.

[2] Faraji J., Metz G.A.S. (2026). Environmental epigenetics: New horizons in redefining biological and health outcomes. Environ. Int..

[3] Kaelin W.G., McKnight S.L. (2013). Influence of Metabolism on Epigenetics and Disease. Cell.

[4] Wilkinson A.L., Zorzan I., Rugg-Gunn P.J. (2023). Epigenetic regulation of early human embryo development. Cell Stem Cell.

[5] Hemberger M., Dean W., Reik W. (2009). Epigenetic dynamics of stem cells and cell lineage commitment: Digging Waddington’s canal. Nat. Rev. Mol. Cell Biol..

[6] Fico A., Fiorenzano A., Pascale E., Patriarca E.J., Minchiotti G. (2019). Long non-coding RNA in stem cell pluripotency and lineage commitment: Functions and evolutionary conservation. Cell. Mol. Life Sci..

[7] Moris N., Pina C., Arias A.M. (2016). Transition states and cell fate decisions in epigenetic landscapes. Nat. Rev. Genet..

[8] Lorzadeh A., Romero-Wolf M., Goel A., Jadhav U. (2021). Epigenetic Regulation of Intestinal Stem Cells and Disease: A Balancing Act of DNA and Histone Methylation. Gastroenterology.

[9] Moltrasio C., Romagnuolo M., Marzano A.V. (2022). Epigenetic Mechanisms of Epidermal Differentiation. Int. J. Mol. Sci..

[10] Mosca S., Ottaviani M., Briganti S., Di Nardo A., Flori E. (2025). The Sebaceous Gland: A Key Player in the Balance Between Homeostasis and Inflammatory Skin Diseases. Cells.

[11] Niemann C., Horsley V. (2012). Development and homeostasis of the sebaceous gland. Semin. Cell Dev. Biol..

[12] Dai W., Qiao X., Fang Y., Guo R., Bai P., Liu S., Li T., Jiang Y., Wei S., Na Z. (2024). Epigenetics-targeted drugs: Current paradigms and future challenges. Signal Transduct. Target. Ther..

[13] Schmidt M., Binder H., Schneider M.R. (2025). The metabolic underpinnings of sebaceous lipogenesis. Commun. Biol..

[14] Okoro O.E., Camera E., Flori E., Ottaviani M. (2023). Insulin and the sebaceous gland function. Front. Physiol..

[15] Szöllősi A.G., Oláh A., Bíró T., Tóth B.I. (2017). Recent advances in the endocrinology of the sebaceous gland. Dermatoendocrinology.

[16] Hou X., Wei Z., Zouboulis C.C., Ju Q. (2022). Aging in the sebaceous gland. Front. Cell Dev. Biol..

[17] Zouboulis C.C., Coenye T., He L., Kabashima K., Kobayashi T., Niemann C., Nomura T., Oláh A., Picardo M., Quist S.R. (2022). Sebaceous immunobiology—Skin homeostasis, pathophysiology, coordination of innate immunity and inflammatory response and disease associations. Front. Immunol..

[18] Lee W.J., Park K.H., Sohn M.Y., Lee W.C., Lee S.J., Kim D.W. (2013). Ultraviolet B irradiation increases the expression of inflammatory cytokines in cultured sebocytes. J. Dermatol..

[19] Clayton R.W., Göbel K., Niessen C.M., Paus R., van Steensel M.A.M., Lim X. (2019). Homeostasis of the sebaceous gland and mechanisms of acne pathogenesis. Br. J. Dermatol..

[20] Hussein L., Perrett C.M. (2021). Treatment of sebaceous gland hyperplasia: A review of the literature. J. Dermatol. Treat..

[21] Dowell-Esquivel C., Lee R., DiCaprio R.C., Nouri K. (2023). Sebaceous carcinoma: An updated review of pathogenesis, diagnosis, and treatment options. Arch. Dermatol. Res..

[22] Makrantonaki E., Ganceviciene R., Zouboulis C. (2011). An update on the role of the sebaceous gland in the pathogenesis of acne. Dermatoendocrinology.

[23] Obata H. (2002). Anatomy and histopathology of human meibomian gland. Cornea.

[24] Treuting P.M., Dintzis S.M., Montine K.S. (2018). Comparative Anatomy and Histology: A Mouse, Rat, and Human Atlas.

[25] Doucet S., Soussignan R., Sagot P., Schaal B. (2009). The secretion of areolar (Montgomery’s) glands from lactating women elicits selective, unconditional responses in neonates. PLoS ONE.

[26] Sheppard J.D., Nichols K.K. (2023). Dry Eye Disease Associated with Meibomian Gland Dysfunction: Focus on Tear Film Characteristics and the Therapeutic Landscape. Ophthalmol. Ther..

[27] Moreno I., Verma S., Gesteira T.F., Coulson-Thomas V.J. (2023). Recent advances in age-related meibomian gland dysfunction (ARMGD). Ocul. Surf..

[28] Shields J.A., Demirci H., Marr B.P., Eagle R.C., Shields C.L. (2005). Sebaceous Carcinoma of the Ocular Region: A Review. Surv. Ophthalmol..

[29] Knop E., Knop N., Millar T., Obata H., Sullivan D.A. (2011). The international workshop on meibomian gland dysfunction: Report of the subcommittee on anatomy, physiology, and pathophysiology of the meibomian gland. Investig. Ophthalmol. Vis. Sci..

[30] Bron A.J., Tripathi R.C., Tripathi B.J., Wolff E., Bron A.J., Tripathi R.C., Tripathi B.J. (1997). Wolff’s Anatomy of the Eye and Orbit.

[31] Verma S., Moreno I.Y., Trapp M.E., Ramirez L., Gesteira T.F., Coulson-Thomas V.J. (2023). Meibomian gland development: Where, when and how?. Differentiation.

[32] Zhu X., Xu M., Portal C., Lin Y., Ferdinand A., Peng T., Morrisey E.E., Dlugosz A.A., Castellano J.M., Lee V. (2025). Identification of Meibomian gland stem cell populations and mechanisms of aging. Nat. Commun..

[33] Butovich I.A. (2017). Meibomian glands, meibum, and meibogenesis. Exp. Eye Res..

[34] Andersen B., Millar S. (2021). Skin epigenetics. Exp. Dermatol..

[35] Allis C.D., Jenuwein T. (2016). The molecular hallmarks of epigenetic control. Nat. Rev. Genet..

[36] Mattei A.L., Bailly N., Meissner A. (2022). DNA methylation: A historical perspective. Trends Genet..

[37] Babenko V.N., Chadaeva I.V., Orlov Y.L. (2017). Genomic landscape of CpG rich elements in human. BMC Evol. Biol..

[38] Moore L.D., Le T., Fan G. (2013). DNA Methylation and Its Basic Function. Neuropsychopharmacology.

[39] Lyko F. (2018). The DNA methyltransferase family: A versatile toolkit for epigenetic regulation. Nat. Rev. Genet..

[40] Gujar H., Weisenberger D.J., Liang G. (2019). The Roles of Human DNA Methyltransferases and Their Isoforms in Shaping the Epigenome. Genes.

[41] Michalak E.M., Burr M.L., Bannister A.J., Dawson M.A. (2019). The roles of DNA, RNA and histone methylation in ageing and cancer. Nat. Rev. Mol. Cell Biol..

[42] Hyun K., Jeon J., Park K., Kim J. (2017). Writing, erasing and reading histone lysine methylations. Exp. Mol. Med..

[43] Greer E.L., Shi Y. (2012). Histone methylation: A dynamic mark in health, disease and inheritance. Nat. Rev. Genet..

[44] Lee M.K., Park N.H., Lee S.Y., Kim T. (2025). Context-Dependent and Locus-Specific Role of H3K36 Methylation in Transcriptional Regulation. J. Mol. Biol..

[45] Yang C., Zhang J., Ma Y., Wu C., Cui W., Wang L. (2020). Histone methyltransferase and drug resistance in cancers. J. Exp. Clin. Cancer Res..

[46] Dimitrova E., Turberfield A.H., Klose R.J. (2015). Histone demethylases in chromatin biology and beyond. EMBO Rep..

[47] Husmann D., Gozani O. (2019). Histone lysine methyltransferases in biology and disease. Nat. Struct. Mol. Biol..

[48] Basavarajappa B.S., Subbanna S. (2021). Histone Methylation Regulation in Neurodegenerative Disorders. Int. J. Mol. Sci..

[49] Zhang Q.J., Liu Z.P. (2015). Histone methylations in heart development, congenital and adult heart diseases. Epigenomics.

[50] Kubo N., Chen P.B., Hu R., Ye Z., Sasaki H., Ren B. (2024). H3K4me1 facilitates promoter-enhancer interactions and gene activation during embryonic stem cell differentiation. Mol. Cell.

[51] LaMere S.A., Thompson R.C., Komori H.K., Mark A., Salomon D.R. (2016). Promoter H3K4 methylation dynamically reinforces activation-induced pathways in human CD4 T cells. Genes Immun..

[52] Shoaib M., Chen Q., Shi X., Nair N., Prasanna C., Yang R., Walter D., Frederiksen K.S., Einarsson H., Svensson J.P. (2021). Histone H4 lysine 20 mono-methylation directly facilitates chromatin openness and promotes transcription of housekeeping genes. Nat. Commun..

[53] Ninova M., Fejes Tóth K., Aravin A.A. (2019). The control of gene expression and cell identity by H3K9 trimethylation. Development.

[54] Cai Y., Zhang Y., Loh Y.P., Tng J.Q., Lim M.C., Cao Z., Raju A., Lieberman Aiden E., Li S., Manikandan L. (2021). H3K27me3-rich genomic regions can function as silencers to repress gene expression via chromatin interactions. Nat. Commun..

[55] Shvedunova M., Akhtar A. (2022). Modulation of cellular processes by histone and non-histone protein acetylation. Nat. Rev. Mol. Cell Biol..

[56] Wapenaar H., Dekker F.J. (2016). Histone acetyltransferases: Challenges in targeting bi-substrate enzymes. Clin. Epigenetics.

[57] Marmorstein R. (2001). Structure of histone acetyltransferases. J. Mol. Biol..

[58] Brownell J.E., Allis C.D. (1996). Special HATs for special occasions: Linking histone acetylation to chromatin assembly and gene activation. Curr. Opin. Genet. Dev..

[59] Parthun M.R. (2012). Histone acetyltransferase 1: More than just an enzyme?. Biochim. Biophys. Acta.

[60] Sun X.J., Man N., Tan Y., Nimer S.D., Wang L. (2015). The Role of Histone Acetyltransferases in Normal and Malignant Hematopoiesis. Front. Oncol..

[61] Milazzo G., Mercatelli D., Di Muzio G., Triboli L., De Rosa P., Perini G., Giorgi F.M. (2020). Histone Deacetylases (HDACs): Evolution, Specificity, Role in Transcriptional Complexes, and Pharmacological Actionability. Genes.

[62] Curcio A., Rocca R., Alcaro S., Artese A. (2024). The Histone Deacetylase Family: Structural Features and Application of Combined Computational Methods. Pharmaceuticals.

[63] Parveen R., Harihar D., Chatterji B.P. (2023). Recent histone deacetylase inhibitors in cancer therapy. Cancer.

[64] English D.M., Lee S.N., Sabat K.A., Baker I.M., Pham T.K., Collins M.O., Cowley S.M. (2024). Rapid degradation of histone deacetylase 1 (HDAC1) reveals essential roles in both gene repression and active transcription. Nucleic Acids Res..

[65] Ishii S. (2021). The Role of Histone Deacetylase 3 Complex in Nuclear Hormone Receptor Action. Int. J. Mol. Sci..

[66] Efthymiadou A., Gu C., Wang C., Wang H., Li Z., Garcia-Irigoyen O., Fan R., Treuter E., Huang Z. (2025). SMRT anchors the HDAC3 corepressor complex to chromatin to regulate inflammatory and metabolic pathways in macrophages. Nucleic Acids Res..

[67] Haberland M., Montgomery R.L., Olson E.N. (2009). The many roles of histone deacetylases in development and physiology: Implications for disease and therapy. Nat. Rev. Genet..

[68] Pu J., Liu T., Wang X., Sharma A., Schmidt-Wolf I.G.H., Jiang L., Hou J. (2024). Exploring the role of histone deacetylase and histone deacetylase inhibitors in the context of multiple myeloma: Mechanisms, therapeutic implications, and future perspectives. Exp Hematol. Oncol..

[69] Pires G.S., Tolomeu H.V., Rodrigues D.A., Lima L.M., Fraga C.A.M., Pinheiro P.d.S.M. (2025). Drug Discovery for Histone Deacetylase Inhibition: Past, Present and Future of Zinc-Binding Groups. Pharmaceuticals.

[70] Shi M.Q., Xu Y., Fu X., Pan D.S., Lu X.P., Xiao Y., Jiang Y.Z. (2024). Advances in targeting histone deacetylase for treatment of solid tumors. J. Hematol. Oncol..

[71] Nemeth K., Bayraktar R., Ferracin M., Calin G.A. (2024). Non-coding RNAs in disease: From mechanisms to therapeutics. Nat. Rev. Genet..

[72] Cech T.R., Steitz J.A. (2014). The Noncoding RNA Revolution—Trashing Old Rules to Forge New Ones. Cell.

[73] Shang R., Lee S., Senavirathne G., Lai E.C. (2023). microRNAs in action: Biogenesis, function and regulation. Nat. Rev. Genet..

[74] O’Brien J., Hayder H., Zayed Y., Peng C. (2018). Overview of MicroRNA Biogenesis, Mechanisms of Actions, and Circulation. Front. Endocrinol..

[75] Kim H., Lee Y.-Y., Kim V.N. (2025). The biogenesis and regulation of animal microRNAs. Nat. Rev. Mol. Cell Biol..

[76] Ivey K.N., Srivastava D. (2015). microRNAs as Developmental Regulators. Cold Spring Harb. Perspect. Biol..

[77] Leung A.K., Sharp P.A. (2010). MicroRNA functions in stress responses. Mol. Cell.

[78] Quah S., Subramanian G., Tan J.S.L., Utami K.H., Sampath P. (2025). MicroRNAs: A symphony orchestrating evolution and disease dynamics. Trends Mol. Med..

[79] Condrat C.E., Thompson D.C., Barbu M.G., Bugnar O.L., Boboc A., Cretoiu D., Suciu N., Cretoiu S.M., Voinea S.C. (2020). miRNAs as Biomarkers in Disease: Latest Findings Regarding Their Role in Diagnosis and Prognosis. Cells.

[80] Fu Z., Wang L., Li S., Chen F., Au-Yeung K.K.-W., Shi C. (2021). MicroRNA as an Important Target for Anticancer Drug Development. Front. Pharmacol..

[81] Yao Q., Chen Y., Zhou X. (2019). The roles of microRNAs in epigenetic regulation. Curr. Opin. Chem. Biol..

[82] Mattick J.S., Amaral P.P., Carninci P., Carpenter S., Chang H.Y., Chen L.-L., Chen R., Dean C., Dinger M.E., Fitzgerald K.A. (2023). Long non-coding RNAs: Definitions, functions, challenges and recommendations. Nat. Rev. Mol. Cell Biol..

[83] Statello L., Guo C.-J., Chen L.-L., Huarte M. (2021). Gene regulation by long non-coding RNAs and its biological functions. Nat. Rev. Mol. Cell Biol..

[84] Zhao Y., Teng H., Yao F., Yap S., Sun Y., Ma L. (2020). Challenges and Strategies in Ascribing Functions to Long Noncoding RNAs. Cancers.

[85] Hezroni H., Koppstein D., Schwartz M.G., Avrutin A., Bartel D.P., Ulitsky I. (2015). Principles of long noncoding RNA evolution derived from direct comparison of transcriptomes in 17 species. Cell Rep..

[86] Gaggi G., Hausman C., Cho S., Badalamenti B.C., Trinh B.Q., Di Ruscio A., Ummarino S. (2025). LncRNAs Ride the Storm of Epigenetic Marks. Genes.

[87] Somasundaram K., Gupta B., Jain N., Jana S. (2022). LncRNAs divide and rule: The master regulators of phase separation. Front. Genet..

[88] Ku D., Yang Y., Kim Y. (2025). RNA-associated nuclear condensates: Where the nucleus keeps its RNAs in check. Mol. Cells.

[89] Noh J.H., Kim K.M., McClusky W.G., Abdelmohsen K., Gorospe M. (2018). Cytoplasmic functions of long noncoding RNAs. Wiley Interdiscip. Rev. RNA.

[90] Aillaud M., Schulte L.N. (2020). Emerging Roles of Long Noncoding RNAs in the Cytoplasmic Milieu. Non-Coding RNA.

[91] Lee H., Zhang Z., Krause H.M. (2019). Long Noncoding RNAs and Repetitive Elements: Junk or Intimate Evolutionary Partners?. Trends Genet..

[92] Chodurska B., Kunej T. (2025). Long non-coding RNAs in humans: Classification, genomic organization and function. Non-Coding RNA Res..

[93] López-Urrutia E., Bustamante Montes L.P., Ladrón de Guevara Cervantes D., Pérez-Plasencia C., Campos-Parra A.D. (2019). Crosstalk Between Long Non-coding RNAs, Micro-RNAs and mRNAs: Deciphering Molecular Mechanisms of Master Regulators in Cancer. Front. Oncol..

[94] Bhattacharjee R., Prabhakar N., Kumar L., Bhattacharjee A., Kar S., Malik S., Kumar D., Ruokolainen J., Negi A., Jha N.K. (2023). Crosstalk between long noncoding RNA and microRNA in Cancer. Cell. Oncol..

[95] Shamloul G., Khachemoune A. (2021). An updated review of the sebaceous gland and its role in health and diseases Part 1: Embryology, evolution, structure, and function of sebaceous glands. Dermatol. Ther..

[96] Andersen H., Ehlers N., Matthiessen M.E. (1965). Histochemistry and development of the human eyelids. Acta Ophthalmol..

[97] Nien C.J., Massei S., Lin G., Liu H., Paugh J.R., Liu C.Y., Kao W.W., Brown D.J., Jester J.V. (2010). The development of meibomian glands in mice. Mol. Vis..

[98] Butovich I.A., Wilkerson A. (2022). Dynamic Changes in the Gene Expression Patterns and Lipid Profiles in the Developing and Maturing Meibomian Glands. Int. J. Mol. Sci..

[99] Geueke A., Niemann C. (2021). Stem and progenitor cells in sebaceous gland development, homeostasis and pathologies. Exp. Dermatol..

[100] Reichenbach B., Classon J., Aida T., Tanaka K., Genander M., Göritz C. (2018). Glutamate transporter Slc1a3 mediates inter-niche stem cell activation during skin growth. Embo J.

[101] Petersson M., Brylka H., Kraus A., John S., Rappl G., Schettina P., Niemann C. (2011). TCF/Lef1 activity controls establishment of diverse stem and progenitor cell compartments in mouse epidermis. Embo J..

[102] Snippert H.J., Haegebarth A., Kasper M., Jaks V., van Es J.H., Barker N., van de Wetering M., van den Born M., Begthel H., Vries R.G. (2010). Lgr6 marks stem cells in the hair follicle that generate all cell lineages of the skin. Science.

[103] Andersen M.S., Hannezo E., Ulyanchenko S., Estrach S., Antoku Y., Pisano S., Boonekamp K.E., Sendrup S., Maimets M., Pedersen M.T. (2019). Tracing the cellular dynamics of sebaceous gland development in normal and perturbed states. Nat. Cell. Biol..

[104] Jensen K.B., Collins C.A., Nascimento E., Tan D.W., Frye M., Itami S., Watt F.M. (2009). Lrig1 expression defines a distinct multipotent stem cell population in mammalian epidermis. Cell Stem Cell.

[105] Olami Y., Zajicek G., Cogan M., Gnessin H., Pe’er J. (2001). Turnover and Migration of Meibomian Gland Cells in Rats’ Eyelids. Ophthalmic Res..

[106] Parfitt G.J., Lewis P.N., Young R.D., Richardson A., Lyons J.G., Di Girolamo N., Jester J.V. (2016). Renewal of the holocrine meibomian glands by label-retaining, unipotent epithelial progenitors. Stem Cell Rep..

[107] Zhang Y., Tchegnon E., Ghotbi E., Chen Z., Moye S.L., He Y., McKay R.M., Le L.Q. (2025). Disruption of Krox20-Notch1 signaling blocks meibomian gland development and homeostasis leading to dry eye disease. Nat. Commun..

[108] Zhu X., Xu M., Owens D.M., Millar S.E. (2025). KRT6A and KRT17 Mark Distinct Stem Cell Populations in the Adult Palpebral Conjunctiva and Meibomian Gland. Cells.

[109] Kutlu Ö., Karadağ A.S., Wollina U. (2023). Adult acne versus adolescent acne: A narrative review with a focus on epidemiology to treatment. An. Bras. Dermatol..

[110] Vasam M., Korutla S., Bohara R.A. (2023). Acne vulgaris: A review of the pathophysiology, treatment, and recent nanotechnology based advances. Biochem. Biophys. Rep..

[111] Sutaria A.H., Masood S., Saleh H.M., Schlessinger J. (2025). Acne Vulgaris. StatPearls.

[112] Bernales Salinas A. (2021). Acne vulgaris: Role of the immune system. Int. J. Dermatol..

[113] Reynolds R.V., Yeung H., Cheng C.E., Cook-Bolden F., Desai S.R., Druby K.M., Freeman E.E., Keri J.E., Stein Gold L.F., Tan J.K.L. (2024). Guidelines of care for the management of acne vulgaris. J. Am. Acad. Dermatol..

[114] Eichenfield D.Z., Sprague J., Eichenfield L.F. (2021). Management of Acne Vulgaris: A Review. JAMA.

[115] Li Y., Hu X., Dong G., Wang X., Liu T. (2024). Acne treatment: Research progress and new perspectives. Front. Med..

[116] Papadimitriou I., Vakirlis E., Sotiriou E., Bakirtzi K., Lallas A., Ioannides D. (2023). Sebaceous Neoplasms. Diagnostics.

[117] Lazar A.J., Lyle S., Calonje E. (2007). Sebaceous neoplasia and Torre-Muir syndrome. Curr. Diagn. Pathol..

[118] Plewig G., Kligman A.M. (1978). Proliferative Activity of the Sebaceous Glands of the Aged. J. Investig. Dermatol..

[119] Fenske N.A., Lober C.W. (1986). Structural and functional changes of normal aging skin. J. Am. Acad. Dermatol..

[120] Farci F., Rapini R.P. (2025). Sebaceous Hyperplasia. StatPearls.

[121] Requena L., Sangüeza O., Requena L., Sangüeza O. (2017). Sebaceous Adenoma and Sebaceoma. Cutaneous Adnexal Neoplasms.

[122] Shalin S.C., Lyle S., Calonje E., Lazar A.J. (2010). Sebaceous neoplasia and the Muir-Torre syndrome: Important connections with clinical implications. Histopathology.

[123] Mulay K., Aggarwal E., White V.A. (2013). Periocular sebaceous gland carcinoma: A comprehensive review. Saudi J. Ophthalmol..

[124] Buitrago W., Joseph A.K. (2008). Sebaceous carcinoma: The great masquerader. Dermatol. Ther..

[125] Owen J.L., Kibbi N., Worley B., Kelm R.C., Wang J.V., Barker C.A., Behshad R., Bichakjian C.K., Bolotin D., Bordeaux J.S. (2019). Sebaceous carcinoma: Evidence-based clinical practice guidelines. Lancet Oncol..

[126] Knackstedt T., Samie F.H. (2017). Sebaceous Carcinoma: A Review of the Scientific Literature. Curr. Treat. Options Oncol..

[127] Narang P., Donthineni P.R., D’Souza S., Basu S. (2023). Evaporative dry eye disease due to meibomian gland dysfunction: Preferred practice pattern guidelines for diagnosis and treatment. Indian J. Ophthalmol..

[128] Kwon J., Moghtader A., Kang C., Bibak Bejandi Z., Shahjahan S., Alzein A., Djalilian A.R. (2025). Overview of Dry Eye Disease for Primary Care Physicians. Medicina.

[129] Nelson J.D., Shimazaki J., Benitez-del-Castillo J.M., Craig J.P., McCulley J.P., Den S., Foulks G.N. (2011). The international workshop on meibomian gland dysfunction: Report of the definition and classification subcommittee. Investig. Ophthalmol. Vis. Sci..

[130] Foulks G.N., Nichols K.K., Bron A.J., Holland E.J., McDonald M.B., Nelson J.D. (2012). Improving awareness, identification, and management of meibomian gland dysfunction. Ophthalmology.

[131] Baudouin C., Messmer E.M., Aragona P., Geerling G., Akova Y.A., Benítez-del-Castillo J., Boboridis K.G., Merayo-Lloves J., Rolando M., Labetoulle M. (2016). Revisiting the vicious circle of dry eye disease: A focus on the pathophysiology of meibomian gland dysfunction. Br. J. Ophthalmol..

[132] Hassanzadeh S., Varmaghani M., Zarei-Ghanavati S., Heravian Shandiz J., Azimi Khorasani A. (2021). Global Prevalence of Meibomian Gland Dysfunction: A Systematic Review and Meta-Analysis. Ocul. Immunol. Inflamm..

[133] Geerling G., Baudouin C., Aragona P., Rolando M., Boboridis K.G., Benítez-del-Castillo J.M., Akova Y.A., Merayo-Lloves J., Labetoulle M., Steinhoff M. (2017). Emerging strategies for the diagnosis and treatment of meibomian gland dysfunction: Proceedings of the OCEAN group meeting. Ocul. Surf..

[134] Ko E.K., Anderson A., D’souza C., Zou J., Huang S., Cho S., Alawi F., Prouty S., Lee V., Yoon S. (2024). Disruption of H3K36 methylation provokes cellular plasticity to drive aberrant glandular formation and squamous carcinogenesis. Dev. Cell.

[135] Driskell I., Oda H., Blanco S., Nascimento E., Humphreys P., Frye M. (2012). The histone methyltransferase Setd8 acts in concert with c-Myc and is required to maintain skin. Embo J..

[136] Li G., Ye Z., Shi C., Sun L., Han M., Zhuang Y., Xu T., Zhao S., Wu X. (2017). The Histone Methyltransferase Ash1l is Required for Epidermal Homeostasis in Mice. Sci. Rep..

[137] Shin H.S., Lee Y., Shin M.H., Cho S.I., Zouboulis C.C., Kim M.K., Lee D.H., Chung J.H. (2021). Histone Deacetylase 1 Reduces Lipogenesis by Suppressing SREBP1 Transcription in Human Sebocyte Cell Line SZ95. Int. J. Mol. Sci..

[138] Shin H.S., Zouboulis C.C., Kim M.K., Lee D.H., Chung J.H. (2022). Minocycline suppresses lipogenesis via inhibition of p300 histone acetyltransferase activity in human SZ95 sebocytes. J. Eur. Acad. Dermatol. Venereol..

[139] Pacella G.N., Kuprasertkul N., Bao L., Huang S., D’souza C., Prouty S.M., Anderson A., Maldonado López A.M., Sinkfield M., Olingou C. (2025). UTX (KDM6A) promotes differentiation noncatalytically in somatic self-renewing epithelia. Proc. Natl. Acad. Sci. USA.

[140] Rinaldi L., Avgustinova A., Martín M., Datta D., Solanas G., Prats N., Benitah S.A. (2017). Loss of Dnmt3a and Dnmt3b does not affect epidermal homeostasis but promotes squamous transformation through PPAR-γ. eLife.

[141] Li J., Jiang T.X., Hughes M.W., Wu P., Yu J., Widelitz R.B., Fan G., Chuong C.M. (2012). Progressive alopecia reveals decreasing stem cell activation probability during aging of mice with epidermal deletion of DNA methyltransferase 1. J. Investig. Dermatol..

[142] Winter M., Moser M.A., Meunier D., Fischer C., Machat G., Mattes K., Lichtenberger B.M., Brunmeir R., Weissmann S., Murko C. (2013). Divergent roles of HDAC1 and HDAC2 in the regulation of epidermal development and tumorigenesis. Embo J..

[143] Hughes M.W., Jiang T.X., Lin S.J., Leung Y., Kobielak K., Widelitz R.B., Chuong C.M. (2014). Disrupted ectodermal organ morphogenesis in mice with a conditional histone deacetylase 1, 2 deletion in the epidermis. J. Investig. Dermatol..

[144] Zhu X., Xu M., Millar S.E. (2024). HDAC1/2 and HDAC3 play distinct roles in controlling adult Meibomian gland homeostasis. Ocul. Surf..

[145] Zhang C., Zhu L., Liu X., Jiang M., Tang Q., Xu F., Lin T., Dong L., He Y. (2021). MicroRNA-3907 promotes the proliferation and migration of sebaceous gland carcinoma of the eyelid by targeting thrombospondin 1. Oncol. Lett.

[146] Bhardwaj M., Sen S., Chosdol K., Sharma A., Pushker N., Kashyap S., Bakhshi S., Bajaj M.S. (2017). miRNA-200c and miRNA-141 as potential prognostic biomarkers and regulators of epithelial-mesenchymal transition in eyelid sebaceous gland carcinoma. Br. J. Ophthalmol..

[147] Zhao H., Yang X., Liu J., Han F., Yang Z., Hu Z., Liu M., Mei Y. (2022). Overexpression of miR-651-5p inhibits ultraviolet radiation-induced malignant biological behaviors of sebaceous gland carcinoma cells by targeting ZEB2. Ann. Transl. Med..

[148] Dull K., Fazekas F., Deák D., Kovács D., Póliska S., Szegedi A., Zouboulis C.C., Törőcsik D. (2021). miR-146a modulates TLR1/2 and 4 induced inflammation and links it with proliferation and lipid production via the indirect regulation of GNG7 in human SZ95 sebocytes. Sci. Rep..

[149] Song X., Fan J., Jia R., Zhou Y., Ge S., Zhang G., Wang H., Fan X. (2019). Identification and regulation pattern analysis of long noncoding RNAs in meibomian gland carcinoma. Epigenomics.

[150] Briganti S., Mosca S., Di Nardo A., Flori E., Ottaviani M. (2024). New Insights into the Role of PPARγ in Skin Physiopathology. Biomolecules.

[151] Jester J.V., Brown D.J. (2012). Wakayama Symposium: Peroxisome proliferator-activated receptor-gamma (PPARγ) and meibomian gland dysfunction. Ocul. Surf..

[152] Kim S.W., Xie Y., Nguyen P.Q., Bui V.T., Huynh K., Kang J.S., Brown D.J., Jester J.V. (2018). PPARγ regulates meibocyte differentiation and lipid synthesis of cultured human meibomian gland epithelial cells (hMGEC). Ocul. Surf..

[153] Rosen E.D., Sarraf P., Troy A.E., Bradwin G., Moore K., Milstone D.S., Spiegelman B.M., Mortensen R.M. (1999). PPAR gamma is required for the differentiation of adipose tissue in vivo and in vitro. Mol. Cell.

[154] Veniaminova N.A., Jia Y.Y., Hartigan A.M., Huyge T.J., Tsai S.Y., Grachtchouk M., Nakagawa S., Dlugosz A.A., Atwood S.X., Wong S.Y. (2023). Distinct mechanisms for sebaceous gland self-renewal and regeneration provide durability in response to injury. Cell Rep..

[155] Cho Y.W., Hong S., Jin Q., Wang L., Lee J.E., Gavrilova O., Ge K. (2009). Histone methylation regulator PTIP is required for PPARgamma and C/EBPalpha expression and adipogenesis. Cell Metab.

[156] Małodobra-Mazur M., Cierzniak A., Kaliszewski K., Dobosz T. (2021). PPARG Hypermethylation as the First Epigenetic Modification in Newly Onset Insulin Resistance in Human Adipocytes. Genes.

[157] Jang M.-K., Kim J.-H., Jung M.H. (2017). Histone H3K9 Demethylase JMJD2B Activates Adipogenesis by Regulating H3K9 Methylation on PPARγ and C/EBPα during Adipogenesis. PLoS ONE.

[158] Yoo Y., Park J.H., Weigel C., Liesenfeld D.B., Weichenhan D., Plass C., Seo D.G., Lindroth A.M., Park Y.J. (2017). TET-mediated hydroxymethylcytosine at the Pparγ locus is required for initiation of adipogenic differentiation. Int. J. Obes..

[159] Zhang D., Tang Z., Huang H., Zhou G., Cui C., Weng Y., Liu W., Kim S., Lee S., Perez-Neut M. (2019). Metabolic regulation of gene expression by histone lactylation. Nature.

[160] Peng X., Du J. (2025). Histone and non-histone lactylation: Molecular mechanisms, biological functions, diseases, and therapeutic targets. Mol. Biomed..

[161] Chen F., Gu M., Xu H., Zhou S., Shen Z., Li X., Dong L., Li P. (2025). Chronic intermittent hypoxia impairs BM-MSC osteogenesis and long bone growth through regulating histone lactylation. J. Transl. Med..

[162] Kulyté A., Kwok K.H.M., de Hoon M., Carninci P., Hayashizaki Y., Arner P., Arner E. (2019). MicroRNA-27a/b-3p and PPARG regulate SCAMP3 through a feed- forward loop during adipogenesis. Sci. Rep..

[163] Kim S.Y., Kim A.Y., Lee H.W., Son Y.H., Lee G.Y., Lee J.W., Lee Y.S., Kim J.B. (2010). miR-27a is a negative regulator of adipocyte differentiation via suppressing PPARgamma expression. Biochem. Biophys. Res. Commun..

[164] Karbiener M., Fischer C., Nowitsch S., Opriessnig P., Papak C., Ailhaud G., Dani C., Amri E.Z., Scheideler M. (2009). microRNA miR-27b impairs human adipocyte differentiation and targets PPARgamma. Biochem. Biophys. Res. Commun..

[165] Lin Q., Gao Z., Alarcon R.M., Ye J., Yun Z. (2009). A role of miR-27 in the regulation of adipogenesis. Febs J..

[166] Lee E.K., Lee M.J., Abdelmohsen K., Kim W., Kim M.M., Srikantan S., Martindale J.L., Hutchison E.R., Kim H.H., Marasa B.S. (2011). miR-130 suppresses adipogenesis by inhibiting peroxisome proliferator-activated receptor gamma expression. Mol. Cell Biol..

[167] Chen L., Chen Y., Zhang S., Ye L., Cui J., Sun Q., Li K., Wu H., Liu L. (2015). MiR-540 as a novel adipogenic inhibitor impairs adipogenesis via suppression of PPARγ. J. Cell. Biochem..

[168] Li H., Xue M., Xu J., Qin X. (2016). MiR-301a is involved in adipocyte dysfunction during obesity-related inflammation via suppression of PPARγ. Pharm. Int. J. Pharm. Sci..

[169] Skårn M., Namløs H.M., Noordhuis P., Wang M.Y., Meza-Zepeda L.A., Myklebost O. (2012). Adipocyte differentiation of human bone marrow-derived stromal cells is modulated by microRNA-155, microRNA-221, and microRNA-222. Stem Cells Dev..

[170] Liu J., Tang T., Wang G.D., Liu B. (2019). LncRNA-H19 promotes hepatic lipogenesis by directly regulating miR-130a/PPARγ axis in non-alcoholic fatty liver disease. Biosci. Rep..

[171] Xie X., Gao M., Zhao W., Li C., Zhang W., Yang J., Zhang Y., Chen E., Guo Y., Guo Z. (2024). LncRNA Snhg3 aggravates hepatic steatosis via PPARγ signaling. eLife.

[172] Cai H., Xu H., Lu H., Xu W., Liu H., Wang X., Zhou G., Yang X. (2022). LncRNA SNHG1 Facilitates Tumor Proliferation and Represses Apoptosis by Regulating PPARγ Ubiquitination in Bladder Cancer. Cancers.

[173] Chen J., Liu Y., Lu S., Yin L., Zong C., Cui S., Qin D., Yang Y., Guan Q., Li X. (2017). The role and possible mechanism of lncRNA U90926 in modulating 3T3-L1 preadipocyte differentiation. Int. J. Obes..

[174] Cooper D.R., Carter G., Li P., Patel R., Watson J.E., Patel N.A. (2014). Long Non-Coding RNA NEAT1 Associates with SRp40 to Temporally Regulate PPARγ2 Splicing during Adipogenesis in 3T3-L1 Cells. Genes.

[175] Divoux A., Karastergiou K., Xie H., Guo W., Perera R.J., Fried S.K., Smith S.R. (2014). Identification of a novel lncRNA in gluteal adipose tissue and evidence for its positive effect on preadipocyte differentiation. Obesity.

[176] Zhang Y., Gu M., Ma Y., Peng Y. (2020). LncRNA TUG1 reduces inflammation and enhances insulin sensitivity in white adipose tissue by regulating miR-204/SIRT1 axis in obesity mice. Mol. Cell. Biochem..

[177] Downie M.M., Kealey T. (2004). Human sebaceous glands engage in aerobic glycolysis and glutaminolysis. Br. J. Dermatol..

[178] Crawford S.O., Hoogeveen R.C., Brancati F.L., Astor B.C., Ballantyne C.M., Schmidt M.I., Young J.H. (2010). Association of blood lactate with type 2 diabetes: The Atherosclerosis Risk in Communities Carotid MRI Study. Int. J. Epidemiol..

[179] Lin X., Xu B., Zheng Y., Coursey T.G., Zhao Y., Li J., Fu Y., Chen X., Zhao Y.E. (2017). Meibomian Gland Dysfunction in Type 2 Diabetic Patients. J. Ophthalmol..

[180] Xu K., Zhang K., Wang Y., Gu Y. (2024). Comprehensive review of histone lactylation: Structure, function, and therapeutic targets. Biochem. Pharmacol..

